# Neurofilament Light Protein Rod Domain Exhibits Structural Heterogeneity

**DOI:** 10.3390/biom14010085

**Published:** 2024-01-09

**Authors:** Victoria V. Nefedova, Sergey Y. Kleymenov, Irina V. Safenkova, Dmitrii I. Levitsky, Alexander M. Matyushenko

**Affiliations:** 1 Research Centre of Biotechnology, Russian Academy of Sciences, 119071 Moscow, Russia; s.yu.kleymenov@gmail.com (S.Y.K.); safenkova@inbi.ras.ru (I.V.S.); levitsky@inbi.ras.ru (D.I.L.); ammatyushenko@mail.ru (A.M.M.); 2 Koltzov Institute of Developmental Biology, Russian Academy of Sciences, 119334 Moscow, Russia

**Keywords:** neurofilament light chain, coiled-coil structure, protein stability, circular dichroism, differential scanning calorimetry, AF4 platform

## Abstract

Neurofilaments are neuron-specific proteins that belong to the intermediate filament (IFs) protein family, with the neurofilament light chain protein (NFL) being the most abundant. The IFs structure typically includes a central coiled-coil rod domain comprised of coils 1A, 1B, and 2, separated by linker regions. The thermal stability of the IF molecule plays a crucial role in its ability for self-association. In the current study, we investigated the thermal stability of NFL coiled-coil domains by analyzing a set of recombinant domains and their fusions (NFL_1B_, NFL_1A+1B_, NFL_2_, NFL_1B+2_, and NFL_ROD_) via circular dichroism spectroscopy and differential scanning calorimetry. The thermal stability of coiled-coil domains is evident in a wide range of temperatures, and thermal transition values (T_m_) correspond well between isolated coiled-coil domains and full-length NFL. NFL_1B_ has a T_m_ of 39.4 °C, and its’ fusions, NFL_1A+1B_ and NFL_1B+2_, have a T_m_ of 41.9 °C and 41.5 °C, respectively. However, in the case of NFL_2_, thermal denaturation includes at least two thermal transitions at 37.2 °C and 62.7 °C. These data indicate that the continuous α-helical structure of the coil 2 domain has parts with varied thermal stability. Among all the NFL fragments, only NFL_2_ underwent irreversible heat-induced denaturation. Together, these results unveil the origin of full-length NFL’s thermal transitions, and reveal its domains structure and properties.

## 1. Introduction

Intermediate filaments (IFs) are part of the cytoskeletal system that consists of three major components in eukaryotic cells. IFs differ from microtubules and microfilaments by family composition, which includes multiple proteins with highly diverse primary structures [1,2,3]. Based on amino-acid sequence homology, the IF family is divided into five major classes (I–V): classes I and II unite acidic and basic keratins, respectively; class III consists of desmin, vimentin, GFAP, and peripherin; class IV includes neurofilament proteins, α-internexin, nestin and synemin, and class V includes lamins [1,3].

Intermediate filaments are presented broadly in various tissues. While some members of the family are widely expressed, others are tissue-specific [1]. However, all intermediate filaments have many common features, including (i) the presence of a central α-helical rod domain in the structure and (ii) an ability to self-assemble. The typical structure of IF is composed of a central rod domain that unites three coiled-coil α-helixes (coil 1A, coil 1B, and coil 2) and N- and C-terminal domains that flank the rod domain and are highly diverse in length and structure [4]. The coiled-coil segments in the rod domain are separated by α-helical linkers L1 (between coils 1A and 1B) and L12 (between coil 1B and coil 2). The primary structure of the α-helical domains is organized as the heptad repeats of amino acids (*abcdefg*)_n_. The amino acids in positions *a* and *d* often have hydrophobic side chains that allow the formation of the hydrophobic core, which enables the self-assembly of α-helical domains into the coiled-coil structure [5]. The IF coiled-coil dimers interact with each other laterally and longitudinally, thus providing the growth of a filament in two directions [4]. The initial step of IF polymerization starts with a tetramer formation [6,7]. The IF tetramers consist of two antiparallel dimers, which are stable in low ionic strength buffers in vitro and were also found in vivo [8].

The IF dimers interact laterally by several modes and form four dimer-to-dimer contacts designated as A_11_, A_12_, A_22_, and A_CN_. In general, three of these modes could be divided into the staggered (A_11_ and A_22_) and unstaggered (A_12_), formed due to different side-by-side antiparallel alignments of the two dimers. The so-called A_11_ tetramer assembly is driven by the alignment of the coil 1B domains of neighboring dimers located antiparallel, approximately in register, by the “anchoring knob—hydrophobic pocket” mechanism [4,9]. The A_22_ mode is formed by the interaction of coil 2 domains of the two neighboring dimers, and the A_12_ mode is formed by the unstaggered antiparallel dimer association. The fourth mode (A_CN_) is assembled through the overlap of the N- and C-ends of two parallel dimers that are longitudinally aligned.

The increase in salt concentration in vitro rapidly leads to the IF polymerization. ULF (unit length filaments) are first formed and then associate into the prolonged assembled filament [10]. In vivo, the self-association of NFs is controlled by post-translational modifications such as phosphorylation of the N-terminal domain [11,12,13].

Most of the IF mature filaments are 10 nm in diameter, while the IF member lamin A/C forms filaments that are only 3.5 nm in diameter [14,15]. For some IFs (such as keratins), the heterodimerization of type I and type II chains is required [16]. Despite the diversity and, to some extent, the uniqueness of IF filaments, when discussing their dimer structures, common features make it possible to transfer the results between protein family members. Most of the principles of IF organization were formulated based on the studies of vimentin from class III [17,18], lamins from class V [19,20], and several keratins [9,16], while here we focus on the class IV member—neurofilaments (NF).

In mature neurons, five members of the IF family are expressed: three NF proteins (light (NFL), medium (NFM), and heavy (NFH) chain proteins), peripherin, and α-internexin [21]. The ratio of the NF proteins in neurons is 7/3/2 per the monomers of NFL/NFM/NFH, respectively [22]. NF proteins are able to form heterodimers, which requires the presence of NFL in each dimer [23,24]. At present, there is a lot of evidence that missense mutations in the *NEFL* gene encode NFL proteins, leading to a neurodegenerative disorder (Charcot–Marie–Tooth neuropathy) [25,26,27,28]. In most cases, these point mutations result in protein aggregation and abnormalities in different stages of NFL self-assembly [25,27].

The thermal stability of different parts of the IF molecule may play an important role in their ability for self-association. For example, coil 1A seems to play an important role in longitudinal polymerization due to its lower thermal stability, which occurs at around 30 °C [29,30]. Importantly, coil 1A of different IFs displays similar values: the melting temperature of vimentin coil 1A is 32 °C, and for coil 1A of lamin A is 33 °C [29]. The strong correlation between the thermal stability of coil 1A and IF self-assembly is proven by the vimentin Y117L mutation, which leads to a pronounced stabilization of coil 1A (up to 71 °C) [29] and to the premature block of filament polymerization on the level of ULFs [31]. Moreover, another vimentin point mutation, K139C, in the linker L1 impaired mature filament formation at 21 °C but did not interfere with the ULF formation [6].

**Figure 1:**
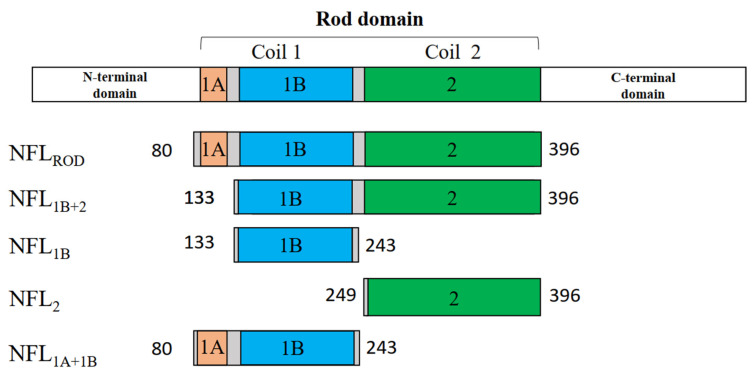
Scheme of the NFL coiled-coil domains and recombinant fragments.

Previously, we studied the thermal stability of the full NFL protein without identifying the correspondence between thermal transitions and elements of the NFL structure [32]. The main aim of the current study was to characterize a relationship between biophysical properties (such as thermal stability) of NFL coiled-coil domains and their possible role in the NFL structure. For this purpose, we developed a panel of recombinant proteins that correspond to the isolated coiled-coil domains of NFL and their combinations (Figure 1). Our approach is similar to “divide-and-conquer”, which is based on the separation of the full IF into smaller parts. This approach was widely accommodated in previous crystallographic studies of IFs and resulted in the coverage of the whole vimentin molecule structure [33,34]. Here, we use this method for studying the NFL coiled-coil domain’s properties both alone and in a more complex structure. While coil 1A of different IFs was widely studied [29,30], the physicochemical properties of the other IFs domains (except its role in IF polymerization) have scarcely been investigated. For proteins containing coiled-coil structures, the distribution of thermal stability across the protein is important. The areas of stability or instability may differ in their roles, either in functional or structural significance. Overall, NFs are less characterized than vimentin, desmin, or keratins; therefore, in our work, we provide a detailed study of all the coiled-coil domains in the NFL molecule.

## 2. Materials and Methods

### 2.1. Protein Expression and Purification

All proteins used in this work were recombinant samples. Full-length NFL (NFL_FL_) (UniProt P07196, Geneva, Switzerland) protein was the product of the *NEFL* gene. The human NFL_FL_ coding sequence was obtained in an EV vector (Cloning Facility, Russia) [32]. All coding sequences of truncated NFL proteins (Table 1, Figure 1) were cloned into restriction sites NdeI and EcoRI of the pET23a (+) vector and had a His-tag on the N-termini.

The following primers were used to obtain different NFL fragments (Table 2): FW133 and REV243 for NFL_1B_; FW80 and REV243 for NFL_1A+1B_; FW133 and REV396 for NFL_1B+2_; FW249 and REV396 for NFL_2_; and FW80 and REV396 for NFL_ROD_. Primer sequences are listed in Table 2. To verify the insert sequence, all NFL fragments were sequenced at the Evrogen company.

Recombinant proteins were expressed in *E. coli* strain C41. The production of proteins was induced by the addition of 1 mM IPTG. NFL_1B_ and NFL_1A+1B_ proteins were purified from the fraction of soluble proteins, while other proteins (NFL_FL_ and other truncated NFL proteins) were purified from the inclusion bodies. NFL_FL_ protein was purified as described previously [32,35]. Inclusion bodies of the truncated NFL forms (NFL_ROD_, NFL_1B+2_, NFL_2_) were solubilized in 50 mM Tris/HCl buffer (pH 8.0) containing 300 mM NaCl, 7 mM β-ME, and 8 M urea, and purified by using metal affinity chromatography on HisTrap HP 5 mL (GE Healthcare, Uppsala, Sweden) column with 15–500 mM imidazole gradient. Purification of NFL_1B_ and NFL_1A+1B_ was carried out in 50 mM Tris/HCl (pH 8.0), 300 mM NaCl buffer with 15–500 mM imidazole gradient. Purified proteins were stored at −80 °C. The purity of the protein preparations was no less than 98%. Protein concentration was determined spectrophotometrically using following E^1%^ values at 280 nm: for NFL_FL_—5.9 cm^−1^; NFL_1A+1B_—2.3 cm^−1^; NFL_1B_—3.5 cm^−1^; NFL_2_—6.8 cm^−1^; NFL_1B+2_—5.3 cm^−1^; and NFL_ROD_—4.4 cm^−1^.

Renaturation of NFL_FL_ proteins and some truncated fragments (NFL_2_, NFL_1B+2_, NFL_ROD_) was achieved by overnight dialysis against a 5 mM HEPES/NaOH buffer, pH 8.0, containing 0.5 mM EGTA, and 2 mM DTT at 4 °C.

### 2.2. Circular Dichroism Spectroscopy (CD)

Far-UV CD spectra of the NF proteins (1 mg/mL) were recorded in the range of 190–280 nm at 5 °C on Chirascan CD spectrometer (Applied Photophysics, Surrey, UK) using cuvettes with an optical path length of 0.02 cm. CD spectra were determined with a 5 s signal registration time for each wavelength using a 1 nm step size. All experiments were carried out in a 5 mM HEPES buffer, pH 8.0, containing 0.5 mM EGTA and 2 mM DTT. Thermal unfolding of NFL fragments was recorded in the range of 5–85 °C with a constant heating rate of 1 °C/min at a wavelength of 222 nm. For CD spectra analysis, the DichroWeb program was used (http://dichroweb.cryst.bbk.ac.uk/html/home.shtml) (London, UK, accessed on 15 January 2022) [36].

### 2.3. Differential Scanning Calorimetry (DSC)

DSC experiments for the NFL proteins were carried out as described previously using a MicroCal VP-Capillary differential scanning mircocalorimeter (Malvern Instruments, Northampton, MA, USA). All experiments were carried out in 5 mM HEPES buffer, pH 8.0, containing 0.5 mM EGTA and 2 mM DTT, and a protein concentration of 2 mg/mL. Protein samples were heated at a constant rate of 1 °C/min in the range of 5–85 °C. The excess heat capacity (ΔC_p_) was calculated using molecular mass for monomers: 12.8 kDa for NFL_1B_, 16.9 kDa for NFL_2_, 30.4 kDa for NFL_1B+2_, 19.2 kDa for NFL_1A+1B_, 36.6 kDa for NFL_ROD_, and 61.5 kDa for NFL_FL_. The computer-assisted deconvolution analysis of the heat absorption curves (i.e., their decomposition into separate thermal transitions (calorimetric domains)) was made using Origin 7.0 software [37].

### 2.4. Fluorescence Experiments

All fluorescence measurements were made on a Cary Eclipse fluorescence spectrophotometer (Varian, Belrose, NSW, Australia). For studies on the temperature-induced denaturation of NFL coil 2, the NFL_2_ and NFL_1B+2_ recombinant proteins were modified by N-(1-pyrene)-iodoacetamide (CHEM-IMPEX, Wood Dale, IL, USA). The modification of the proteins was carried out in 150 mM Na-phosphate buffer, pH 7.4, containing 8 M urea. Before the modification of Cys residues, the proteins were reduced by adding 10 mM DTT, then purified from excess DTT using NAP10 columns (GE Healthcare, Altrincham, UK). The N-(1-pyrene)-iodoacetamide dissolved in dimethylformamide was added to reduced proteins in a 10-fold molar excess. The modification was conducted overnight at 30 °C in the dark. The reaction was stopped by the addition of 5 mM DTT and centrifugation at 14,500× *g* for 15 min. The labeled NFL (Pyr-NFL_2_ or Pyr-NFL_1B+2_) was purified on a NAP10 column. Prior to the experiment, the labeled proteins were dialyzed overnight at 4 °C against 5 mM HEPES/NaOH buffer, pH 8.0, containing 0.5 mM EGTA. The concentration of pyrene attached to the Pyr-NFL_2_ or Pyr-NFL_1B+2_ was determined spectrophotometrically using ε at 344 nm equal to 22,000 M^−1^ cm^−1^. The total protein concentration in the samples was determined by SDS-PAGE. The final ratio of modification was 0.7–0.8 mole pyrene per mole NFL. The spectra of the modified Pyr-NFL_2_ or Pyr-NFL_1B+2_ were recorded at a concentration of 0.8 mg/mL by using fluorescence excitation wavelength 344 nm and fluorescence emission in the range of 350–600 nm. The modified proteins were subjected to heating with a constant rate of 1 °C/min, and fluorescence emission at 385 and 480 nm was registered. Tryptophan fluorescence of NFL_2_ and NFL_1B+2_ was recorded at a 1 mg/mL concentration by fluorescence excitation at 295 nm and measured in the range 300–500 nm.

### 2.5. Asymmetrical Flow Field-Flow Fractionation Coupled with UV/VIS Detector

The molecular size and oligomeric state of the NFL_1B_ fragments were determined by using the AF4 platform that included the Wyatt Eclipse 3+ Separation System with Eclipse Long Channel (Wyatt Technology, Goleta, CA, USA), 1260 Infinity LC System (Isocratic Pump, Autosampler, Variable Wavelength Detector) (Agilent Technologies, Santa Clara, CA, USA) [38]. The experiments were performed in 5 mM HEPES buffer, pH 8.0, containing 0.5 mM EGTA and 2 mM DTT. The samples (1–5 mg/mL) were analyzed at a wavelength of 280 nm. The liquid flow program is presented in Table S1. The data were collected and analyzed with ChemStation v.B.04.03 (Agilent Technologies, Santa Clara, CA, USA) and Astra v.6.1.7.17 (Wyatt Technology, Goleta, CA, USA) software.

### 2.6. Size Exclusion Chromatography (SEC) of NFL Fragments

The oligomeric state of all NFL fragments was obtained from SEC data coupled with chemical cross-linking by glutaraldehyde (TED PELLA, Redding, CA, USA). The SEC was performed in 5 mM HEPES buffer, pH 8.0, containing 0.5 mM EGTA and 2 mM DTT. 250 µL of samples containing 2 mg/mL of proteins was applied on a Superose 6 Increase 10/300 GL column (GE Healthcare, Uppsala, Sweden). The protein elution was performed at 1 mL/min. The chemical cross-linking of samples collected after SEC was carried out in 5 mM HEPES buffer, pH 8.0, containing 0.5 mM EGTA by the addition of an aqueous solution of glutaraldehyde (TED PELLA, Redding, CA, USA). The glutaraldehyde concentrations were 0.008% and 0.024%. The cross-linking was performed at 30 °C for 60 min, and the samples were analyzed by SDS-PAGE in 12.5% gel.

## 3. Results

### 3.1. Expression and Purification of the Recombinant NFL Domains

**Figure 2:**
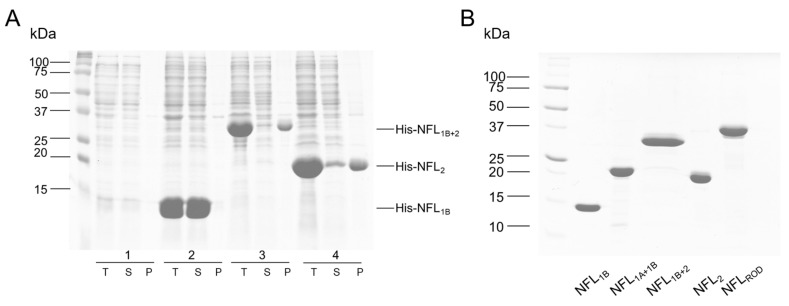
Expression and purification of the NFL fragments. (**A**) Expression of NFL_1B+2_, NFL_2_, and NFL_1B_ in *E.coli* C41 strain. T—total protein of *E. coli*, S—soluble proteins fraction, P—insoluble proteins fraction obtained after centrifugation of the lysate at 18,000× *g*. Lane 1—bacterial lysate before the induction with IPTG, lanes 2–4—bacterial lysate 12 h after the induction with IPTG. (**B**) SDS-PAGE of the purified NFL fragments.

The isolated coiled-coil domains of NFL and their different combinations were obtained in a bacterial expression system. During this step, several differences in their properties were observed. NFL_1B_ and NFL_1A+1B_ recombinant proteins were predominantly expressed in the fraction of soluble bacterial proteins, while NFL_1B+2_, NFL_2_, and NFL_ROD_ (as well as NFL_FL_) were found in the fraction of insoluble bacterial proteins (Figure 2A). The purity of the obtained recombinant proteins was no less than 98% (Figure 2B).

### 3.2. Thermal Stability of the NFL Domains

#### 3.2.1. NFL Rod Domain

**Figure 3:**
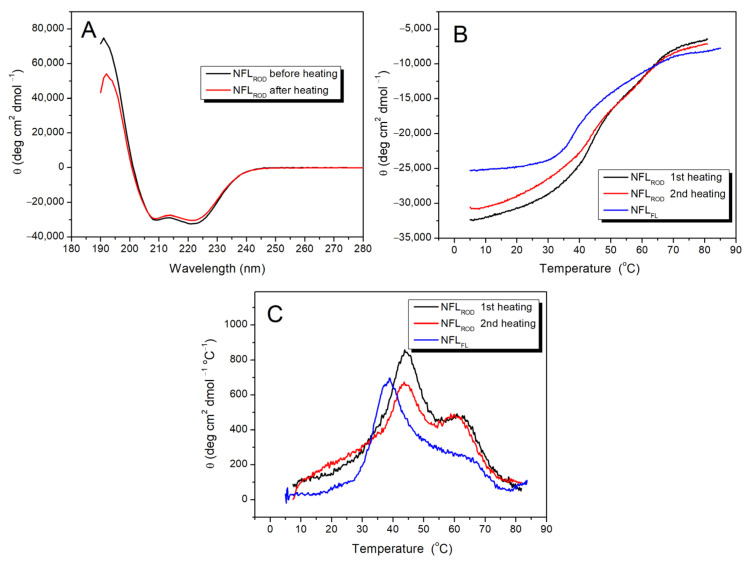
Thermal stability of the NFL_ROD_ domain. (**A**) CD spectra of the NFL_ROD_ protein were recorded at 5 °C before and after heating. (**B**) CD melting curves of NFL_ROD_ recorded at 222 nm and compared with that for the full-length NFL (NFL_FL_). (**C**) The first derivative analysis of the CD melting curves is presented in panel (**B**).

The IF rod domain is composed of full-length coil 1 and coil 2, while full-length NFL also contains non-helical N- and C-termini (Figure 1). The CD spectra of NFL_ROD_ demonstrated 80% of α-helical content with characteristics for this type of structure negative maxima at 208 and 222 nm (Figure 3A). The thermal denaturation of NFL_ROD_ (Figure 3B) and the denaturation of full-length NFL [32] were reversible. Applying the first derivative mathematical analysis of melting curves, we identified two distinguished thermal transitions at 44 °C and 61 °C (Figure 3C). The overall shape of the first derivative of the melting curve for the full-length NFL (NFL_FL_) denaturation was similar to that of the NFL_ROD_. However, the low-temperature thermal transition of NFL_FL_ shifted by almost 4 °C (from 44 °C to 38.2 °C) in comparison with that of NFL_ROD_, while the second thermal transition was less pronounced than that of the rod domain (Figure 3C).

**Figure 4:**
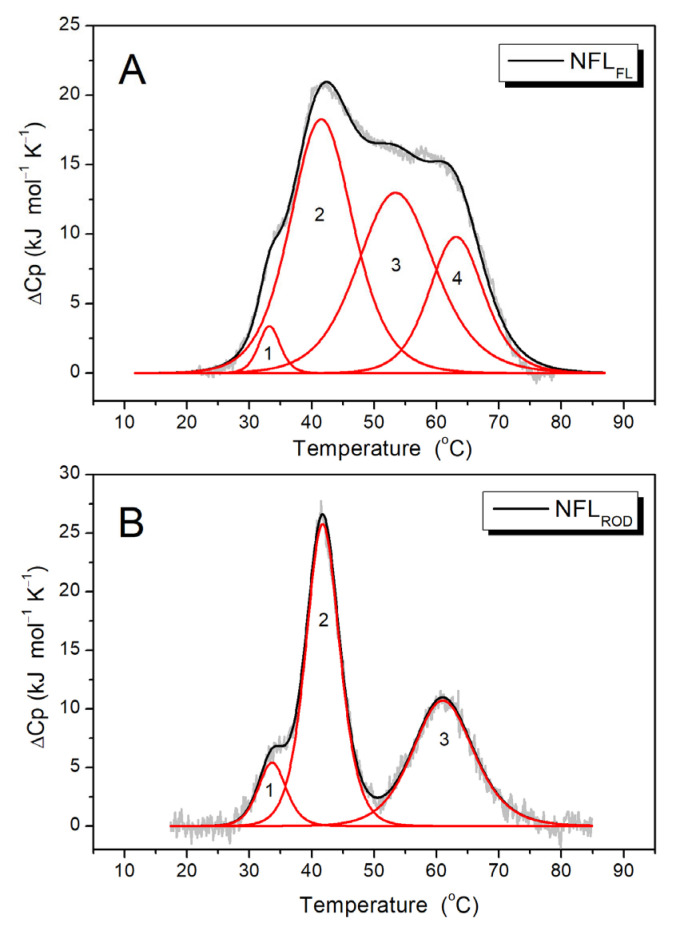
Temperature dependences of excess heat capacity (ΔC_p_) of NFL_FL_ (**A**) and NFL_ROD_ (**B**) obtained by DSC. Experimental DSC curves (after subtraction of instrumental and chemical baselines) are shown by solid grey lines, solid black lines obtained from fitting the data to the non-two-state model, and red lines represent the calorimetric domains (individual thermal transitions) obtained by the deconvolution analysis. The calculated T_m_ and ΔH_cal_ are presented in Table 3. NFL_FL_—full-length NFL, NFL_ROD_—NFL rod domain.

While CD spectroscopy allows measurement of the melting of α-helical coiled-coil domains, the DSC method provides accurate temperature dependences of excess heat capacity (ΔC_p_), which in the case of the reversible denaturation of protein could be decomposed into several individual thermal transitions (calorimetric domains) (Figure 4). The NFL_ROD_ domain has three calorimetric domains at 33.7 °C (domain 1), 41.8 °C (domain 2), and 61.1 °C (domain 3) (Figure 4B), with the highest enthalpy at the domain 2. For the full-length NFL tetramer, four calorimetric domains with a maximum transition rate at 33.2, 41.7, 53.6, and 63.2 °C were found (Figure 4A) [32]. The thermal transitions of domains 1 and domain 2 of NFL_FL_ and NFL_ROD_ correlate well with each other. The domain 3 of NFL_ROD_ and domain 4 of the full-length NFL also seem to be the same. However, domain 3 of NFL_FL_ did not match any of NFL_ROD_ domain.

#### 3.2.2. NFL Coiled-Coil Fragments Containing Coil 1B (NFL_1B_, NFL_1A+1B_, and NFL_1B+2_)

**Figure 5:**
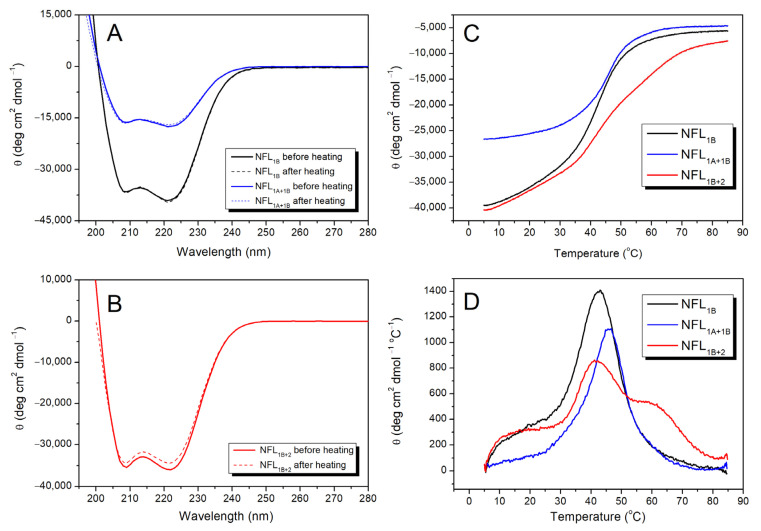
Thermal stability of NFL_1B_, NFL_1A+1B_, and NFL_1B+2_ proteins. (**A**) CD spectra of NFL_1B_ and NFL_1A+1B_ were recorded at 5 °C before and after heating. (**B**) CD spectra of NFL_1B+2_ were recorded at 5 °C before and after heating. (**C**) CD melting curves of NFL_1B_, NFL_1A+1B_, and NFL_1B+2_ were recorded at 222 nm. (**D**) The first derivative analysis of the CD melting curves is presented in panel (**C**).

**Figure 6:**
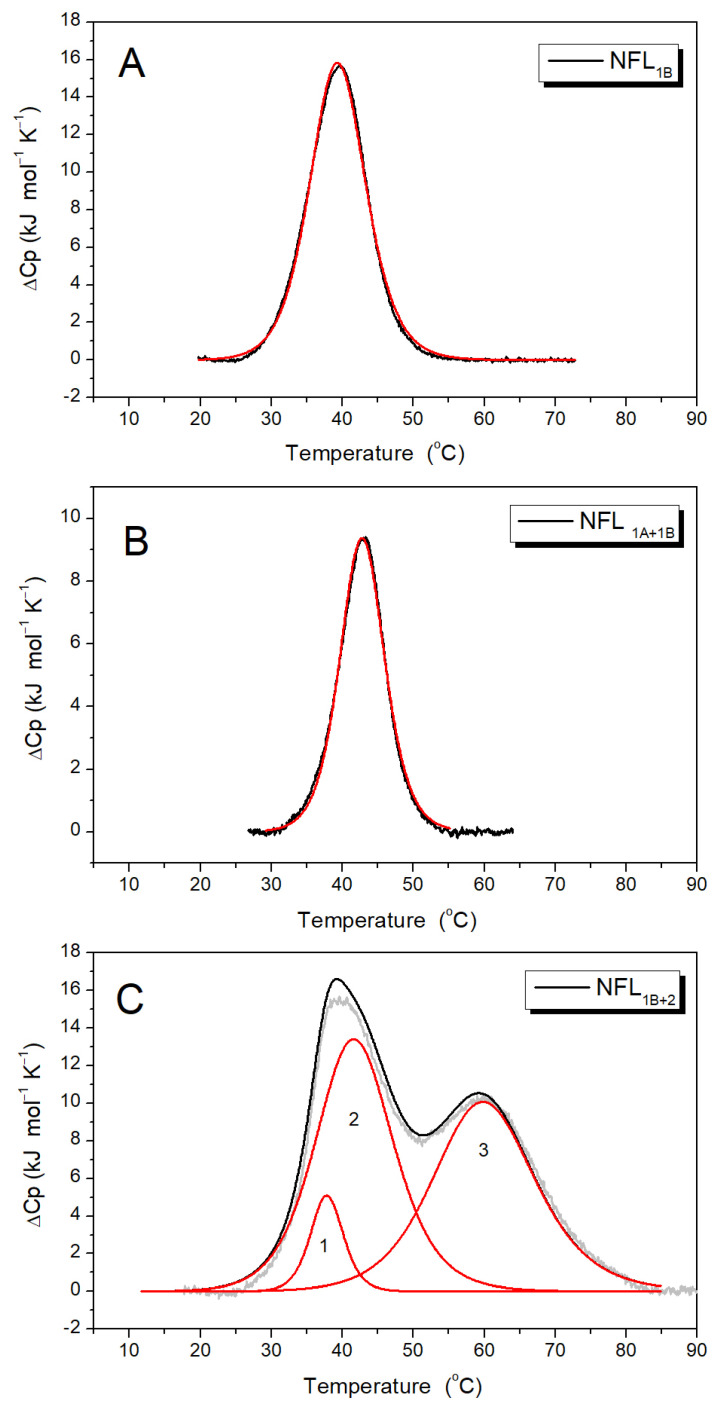
Temperature dependences of excess heat capacity (ΔC_p_) of the NFL_1B_ (**A**), NFL_1A+1B_ (**B**), and NFL_1B+2_ (**C**) proteins obtained by DSC. The experimental DSC curves (after subtraction of instrumental and chemical baselines) are shown by solid grey lines, solid black lines obtained from fitting the data to the non-two-state model, and red lines represent the calorimetric domains (individual thermal transitions) obtained by the deconvolution analysis. The calculated T_m_ and ΔH_cal_ are presented in Table 3. NFL_1B_—NFL coil 1B domain, NFL_1A+1B_—NFL coil 1A and coil 1B domains, NFL_1B+2_—NFL coil 1B and coil 2 domains.

The CD spectra of NFL_1B_, NFL_1A+1B_, and NFL_1B+2_ have two negative maxima at 208 and 222 nm (Figure 5A,B). However, the content of α-helix differs between them. While NFL_1B_ and NFL_1B+2_ contain about 80% of α-helix, NFL_1A+1B_ shows almost a 3-fold decrease in α-helix content in comparison. The thermal melting curves were registered at 222 nm within a range of temperatures from 5 to 85 °C (Figure 5C). The first derivative analysis of a melting curve revealed a single thermal transition peak at ~42 °C for NFL_1B_ and at ~46 °C for NFL_1A+1B_ (Figure 5D). The thermal denaturation of these NFL fragments was fully reversible (Figure 5A,B), thus making possible the computer-assisted deconvolution analysis of DSC melting curves of these proteins (Figure 6A,B; Table 3). The temperature dependence of the excess heat capacity of NFL_1B_ presented a symmetrical peak with a single calorimetric domain with a maximum of 39.4 °C (Figure 6A). The NFL_1A+1B_ fragment also demonstrated the single domain at 42.8 °C (Figure 6B). The thermal denaturation of NFL_1B+2_ was also fully reversible (Figure 5B). This fragment demonstrated the thermal transition at ~42 °C and the high-temperature shoulder at ~60 °C upon CD studies (Figure 5D). In good agreement with CD data, similar thermal transitions (calorimetric domains 2 and 3) at 41.5 °C and 59.6 °C were revealed upon deconvolution of the DSC curve for NFL_1B+2_ (Figure 6C; Table 3).

#### 3.2.3. NFL Coil 2

**Figure 7:**
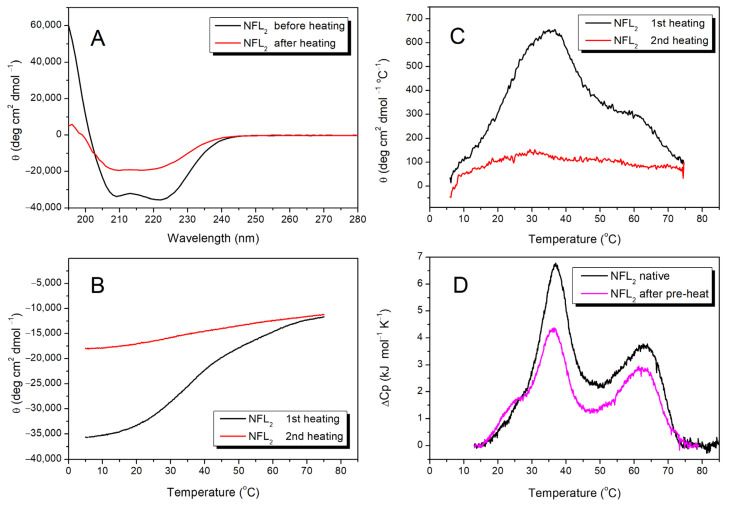
Thermal stability of NFL_2_. (**A**) CD spectra of NFL_2_ protein were recorded at 5 °C before and after heating. (**B**) CD melting curves were recorded at 222 nm. (**C**) First derivative analysis of the CD melting curves presented in panel (**B**). (**D**) Temperature dependence of excess heat capacity (ΔC_p_) of the NFL_2_ protein obtained by DSC. Experimental DSC curves (after subtraction of instrumental and chemical baselines) are shown, and the calculated T_m_ and ΔH_cal_ are presented in Table 3. The black line represents the denaturation of the native NFL_2_ protein heated from 15 to 85 °C. The magenta line represents the denaturation of the pre-heated protein. The first heating was from 15 °C to 47 °C; then the protein was subjected to the second heating from 15 to 85 °C.

Previously, we studied the stability of NFL coil 2 in a fusion with the 1B domain (NFL_1B+2_) (Figure 5B–D and Figure 6C). To investigate the properties of the coil 2 domain in more detail, we produced the NFL_2_ fragment, which consists of this domain alone (see Figure 1). The initial CD spectrum of NFL_2_ showed a negative maxima at 208 and 222 nm, characteristic of the coiled-coil structure (Figure 7A). Heat-induced denaturation measured by CD showed that the NFL_2_ melting curve has several transitions: the main peak at ~35 °C and a shoulder at higher temperatures around 60 °C (Figure 7C). However, the thermal denaturation of NFL_2_ was not fully reversible (Figure 7A–C), indicating that this fragment might have a tendency to aggregate or some other properties hindering its reversible unfolding. Taking into account that the thermal denaturation of NFL_ROD_ and NFL_1B+2_ was fully reversible (Figure 3A,B and Figure 5B), one can suppose that coil 2 in the NFL structure might be stabilized by the interaction with coil 1B or might form complexes with the other NFL domains and, thus, these complexes demonstrated a fully reversible thermal denaturation.

Since the denaturation of NFL_2_ was irreversible, we were unable to apply deconvolution of DSC curves for this protein. However, in good agreement with CD data (Figure 7C), the DSC melting curve for NFL_2_ was presented as two peaks with maxima of thermal transitions at 37.2 °C and 62.7 °C (Figure 7D).

Among all the NFL fragments studied in this work, only the NFL_2_ protein underwent irreversible heat-induced denaturation. The melting curve of NFL_2_ had two distinguished thermal transitions (Figure 7C,D), which allowed us to investigate which one had caused the irreversible melting. In order to do that, we applied a two-step heating protocol: first heating from 15 to 47 °C (the temperature range corresponds to the first peak, Figure 7D), with subsequent cooling in the DSC cell, and the second heating from 15 to 85 °C (Figure S1). The first thermal transition was fully reversible (Figure S1), and the second heating (Figure 7D, magenta line) of NFL_2_ triggered two thermal transitions. Therefore, we assume that the second thermal transition that occurs at 62.7 °C (Figure 7D) is the one responsible for the irreversible NFL_2_ denaturation.

#### 3.2.4. Fluorescence Studies of the NFL Fragments

**Figure 8:**
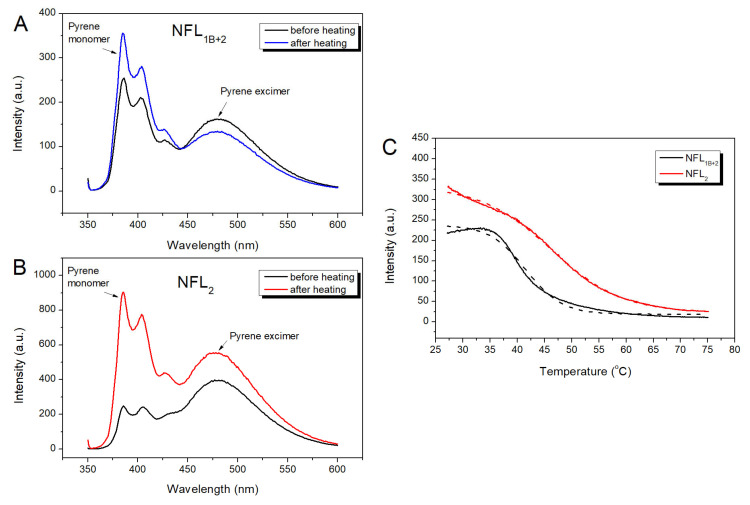
Temperature dependences of the NFL_1B+2_ and NFL_2_ denaturation labeled by pyrene attached to Cys322 in the coil 2. (**A**,**B**) Fluorescence spectra of Pyr-NFL_1B+2_ (**A**) and Pyr-NFL_2_ (**B**) measured before the heat-induced denaturation and after the renaturation. Spectra were registered at 15 °C. (**C**) Temperature dependence of excimer fluorescence of pyrene label in Pyr-NFL_1B+2_ and Pyr-NFL_2_ (solid lines). The curves were fitted by a Boltzmann sigmoidal decay function (dotted lines).

**Figure 9:**
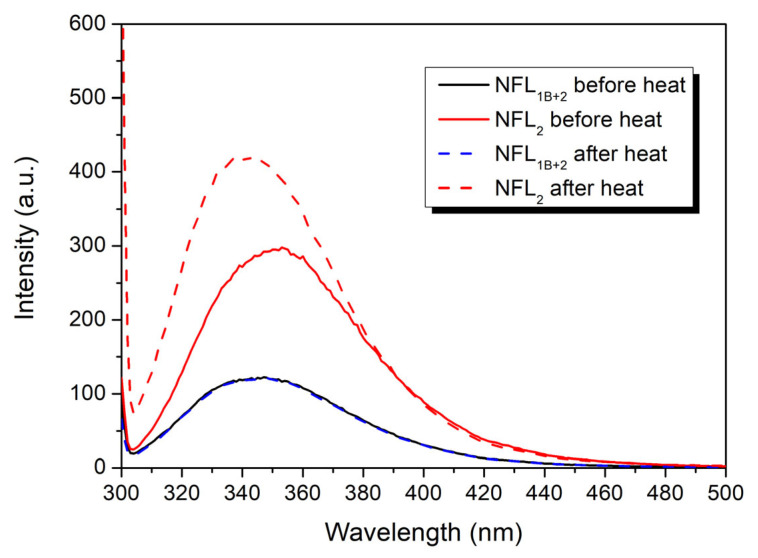
Spectra of tryptophan fluorescence of NFL_1B+2_ and NFL_2_ before and after the heat-induced denaturation.

For the identification of thermal transitions revealed by CD and DSC studies, we applied a fluorescence measurement approach (Figure 8 and Figure 9). The NFL molecule has a single Trp residue (at position 279) and a single Cys residue (at position 322). Based on vimentin structure [18], vimentin and NFL sequences alignment [39], and coiled-coil predictions, NFL position 322 corresponds to a position *d* in the heptad repeats. Trp279 is located at the beginning of the coil 2 domain, which corresponds to the hendecad repeats (*abcdefghijk*)_n_ [17,40,41,42], and its position could not be simply predicted by the ordinary coiled-coil analyzers.

The fluorescence spectra of pyrene-labeled Pyr-NFL_2_ or Pyr-NFL_1B+2_ presented both peaks of the pyrene monomer and excimer fluorescence (Figure 8A,B), which indicated that the Cys322 position is located in the core. However, the ratio of pyrene excimer fluorescence to that of its monomer was higher in NFL_2_. Both Pyr-NFL_2_ and Pyr-NFL_1B+2_ were subjected to heating and subsequent cooling (Figure 8). In the case of Pyr-NFL_1B+2_, the fluorescence spectra measured before and after heating uncovered some changes, namely, a slight decrease in pyrene excimer fluorescence and an increase in pyrene monomer fluorescence (Figure 8A). In the case of the Pyr-NFL_2_ fragment, the fluorescence spectra obtained before and after heating were dramatically different; in particular, the ratio of pyrene monomer/excimer fluorescence shifted (Figure 8B). Before heating, the excimer fluorescence was 1.8 times higher than that of the monomer, while after heating, pyrene monomer fluorescence became 2 times higher than that of the excimer. Such extreme changes in the fluorescence indicated that the NFL_2_ fragment alone is not able to form the right conformation after the heat-induced denaturation. The pyrene excimer fluorescence decreased upon the thermal denaturation (Figure 8C), and a half-maximal decrease for Pyr-NFL_1B+2_ occurred at 41.6 ± 0.1 °C, while for Pyr-NFL_2_ it was 46.1 ± 0.1 °C. Such differences also indicate that coil 2 alone adopts a conformation that differs from the one it acquires as a part of NFL_1B+2_. This suggestion is also confirmed by tryptophan fluorescence experiments (Figure 9). The NFL_2_ and NFL_1B+2_ proteins Trp fluorescence spectra showed similar maxima at 353 and 347 nm, respectively. These maxima of the spectra correspond to the Trp residues, which are located on the proteins’ surface and exposed to an aqueous environment [43]. Therefore, tryptophan fluorescence of NFL is not useful for the investigation of the NFL thermal stability. However, we compared the Trp fluorescence spectra of NFL_2_ and NFL_1B+2_ proteins before and after heating (Figure 9) and found that while NFL_1B+2_ fluorescence spectra before and after heating repeat each other completely, the Trp fluorescence spectra of NFL_2_ after heating has changed its maxima, indicating that Trp residue in the NFL_2_ is in another environment after heating compared to before heating. The data obtained from the fluorescence of the pyrene label attached to Cys amino acid residue and the Trp fluorescence both supported the CD and DSC (Figure 7) data and exemplified the irreversible heat-induced denaturation of NFL_2_.

#### 3.2.5. Oligomerization of NFL Fragments

**Figure 10:**
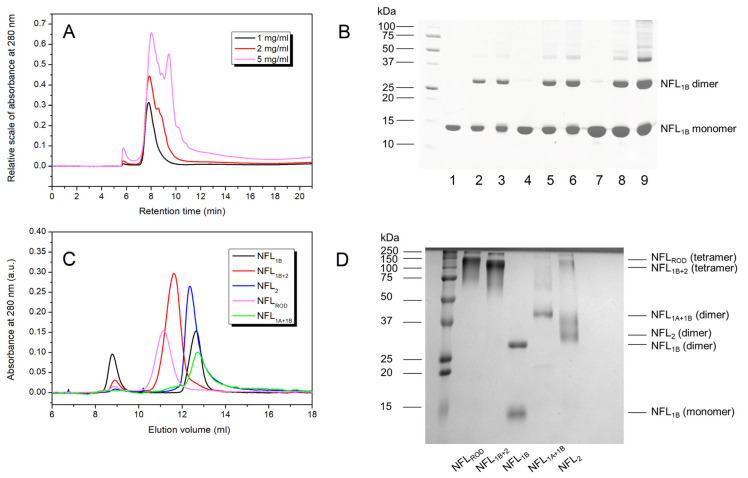
Oligomerization of NFL_1B_. (**A**) AF4–UV/VIS fractograms of NFL_1B_ loaded in different concentrations (1, 2, and 5 mg/mL). (**B**) Chemical cross-linking of NFL_1B_ by glutaraldehyde. Lanes 1, 4, and 7 show the control samples of NFL_1B_ without glutaraldehyde at concentrations 1, 2, and 5 mg/mL, respectively. Lanes 2, 5, and 8 show NFL_1B_ cross-linked by 0.008% of glutaraldehyde at protein concentrations of 1, 2, and 5 mg/mL, respectively. Lanes 3, 6, and 9 show NFL_1B_ cross-linked by 0.024% of glutaraldehyde at protein concentrations of 1, 2, and 5 mg/mL, respectively. (**C**) SEC profiles of NFL fragments (2 mg/mL) uploaded to Superose 6 Increase 10/300 GL column. (**D**) Chemical cross-linking of NFL fragments by 0.024% glutaraldehyde. The molecular masses of NFL fragments were determined by SDS-PAGE.

To investigate the oligomeric state of the NFL fragments containing coil 1B, we used the AF4 platform (Figure 10A) and chemical cross-linking by glutaraldehyde (Figure 10B). In the range of protein concentrations from 1 to 5 mg/mL, NFL_1B_ showed several distinguished peaks with different retention times (Figure 10A). At 1 mg/mL, the NFL_1B_ sample presented the single peak with a retention time of 7.8 min. This NFL_1B_ form is also present in a sample containing 2 mg/mL of protein; meanwhile, this sample contains larger particles as well. These particles separate at the concentration of 5 mg/mL as a peak with a retention time of 9.3 min. The cross-linking demonstrated that at 1 and 2 mg/mL, NFL_1B_ forms dimers in solution, while larger oligomers are also present at 5 mg/mL (Figure 10B). However, cross-linking did not reveal the real stoichiometry of these complexes. 

The oligomeric state of the NFL fragments was also studied by SEC coupled with chemical cross-linking by glutaraldehyde (Figure 10C,D). The proteins were uploaded at concentrations of 2 mg/mL (Figure 10C), and after chromatography, the samples from each peak were cross-linked (Figure 10D). The NFL_ROD_ was eluted with a single major peak (magenta line; Figure 10C). According to SDS-PAGE, after cross-linking, the molecular weight of this fragment corresponds to NFL_ROD_ tetramers (Figure 10D). The same results were obtained for the NFL_1B+2_ fragment (Figure 10C; red line), which also was found to be a tetramer in solution. The others (NFL_1A+1B_ and NFL_2_) exhibit dimeric structures (Figure 10D). Unlike all other fragments, for NFL_1B_, after cross-linking by glutaraldehyde, we also observed monomeric forms (Figure 10B,D); however, the SEC profile showed two peaks that might correspond to very large particles (black line; Figure 10C; elution volume 8.8 mL) and a smaller one (black line; Figure 10C; elution volume 12.6 mL). We assumed that the NFL_1B_ monomers observed on SDS-PAGE (Figure 10B,D) are due to incomplete cross-linking of highly ordered coil 1B. Therefore, we might conclude that NFL_1B_ also forms dimers.

## 4. Discussion

In the present work, we studied the physicochemical properties of the NFL rod domain on the set of isolated recombinant coiled-coil domains (1B and 2) and fusion constructs of different domains, such as 1A+1B, 1B+2, and full rod domain (1A+1B+2) (Figure 1). At the stage of protein expression, we observed the first differences in their properties. NFL_1B_ and NFL_1A+1B_ were purified directly from the soluble fraction of bacterial proteins. At the same time, the expression of NFL_1B+2_, NFL_2_, and NFL_ROD_ in bacteria caused the formation of inclusion bodies, and these recombinant proteins were purified by using buffers containing 8M urea and required further renaturation procedure by dialysis (Figure 2A). All the NFL fragments contained α-helical structure (Figure 3, Figure 5 and Figure 7). However, the NFL_1A+1B_ fragment, which corresponds to the full coil 1 showed almost 3-fold less α-helical content than NFL_ROD_, NFL_1B+2_, NFL_2_, and NFL_1B_. Taking into account that the coil 1 region is also composed of coiled-coils based on the available atomic structures, that seems intriguing. Previously, this phenomenon was also observed for vimentin coil 1 fragment, which has ~30% of α-helical content, compared to isolated coil 1B, coil 2, or full rod domain with about 80% of α-helical content [33]. Such decreases in α-helical content in full coil 1, which includes both 1A and 1B domains, might be due to the coil 1A part. Wild-type vimentin and lamin isolated coil 1A domains contain 55% and 45% of α-helixes, respectively [29]. The ability of coil 1A to form α-helix could be improved by introducing mutations F114V (in position *a* of the heptad) and Y117L (in position *d* of the heptad). These substitutions lead to the increase in coil 1A α-helical content to 69% and 97%, respectively [29]. The potential of peptide to form α-helical structure also strongly depends on the solution composition. For example, either a change in the pH of a buffer or the addition of trifluoriethanol might increase α-helical formation in short peptides from desmin [44]. Coil 1A of vimentin also showed tunable monomer/dimer equilibrium, which depends on the protein concentration. At 0.2 mg/mL, this peptide was found to be a monomer (4.4 kDa), while at 0.8 mg/mL, it self-associated and formed dimers (9 kDa). This equilibrium could be switched to dimer formation not only by increasing protein concentration but also by F114V and Y117L mutations [29].

Previous studies of the thermal unfolding of NFL_FL_ tetramers showed that the DSC curve can be decomposed into four thermal transitions with maxima at 33.2, 41.7, 53.6, and 63.2 °C (Table 3) [32]. The truncated NFL rod domain, in turn, has three thermal transitions at 33.7, 41.8, and 61.1 °C (Figure 4B; Table 3). In both cases, one of the main calorimetric domains was domain 2, with a temperature maximum of 41.7 °C. In the current work, four recombinant coiled-coil fragments have domains with a similar maximum of thermal transitions: NFL_1B_ (single domain with T_m_ = 39.4 °C), NFL_1B+2_ (domain 2 with T_m_ = 41.5 °C), NFL_1A+1B_ (single domain with T_m_ = 41.9 °C), and NFL_ROD_ (domain 2 with T_m_ = 41.8 °C) (Table 3), and all of which have coil 1B in common. Such good correspondence of the thermal transition of coil 1B in the form of a recombinant protein NFL_1B_ and as a part of the full molecule might indicate that its structure remains the same in the tetramer NFL molecule and in an isolated form in solution. The symmetry of the NFL_1B_ fragment thermal denaturation peak (Figure 6A) also indicates that NFL coil 1B is homogeneous throughout its length. It is also important to mention that NFL_1B_ protein was not subjected to urea-containing solutions during purification, while NFL_1B+2_, NFL_ROD_, and NFL_FL_ were. The good correspondence between the T_m_ value for coil 1B denaturation in all these preparations indicated that the successful refolding was achieved after all denaturation agents they may have been subjected to. However, the neighboring domains, as well as their interactions, might affect the stability of coil 1B.

In previous works, the thermal stability of the vimentin rod domain was examined by using short coiled-coil fragments of approximately 50 amino acids in length [30]. In that study, six fragments, which covered the rod domain of vimentin from the beginning of coil 1A to the end of coil 2, were used. The lower stability in the vimentin rod domain was shown for coil 1A and the first third of coil 2 [29,30]. Moreover, the stabilization of coiled-coil segment by N- or C-caps would seriously affect their thermal stability, and in some cases, that difference reached 23 °C. In the present study, we observed the phenomenon wherein the T_m_ of the isolated coiled-coil fragment versus its fusion with other domains was slightly different. For the coil 1B domain, for example, this difference in the T_m_ value was around 2.5 °C (see Table 3).

The important aspect that needed clarification was the oligomeric state of the NFL fragments. By using the AF4 platform, SEC, and chemical cross-linking, we have shown that at 2 mg/mL, the NFL_1B_, NFL_1A+1B_, and NFL_2_ fragments are dimers (Figure 10), and the NFL_1B+2_ and NFL_ROD_ fragments are tetramers (Figure 10C,D). Our data are in good correspondence with data obtained on the other IFs. In earlier works, the molecular mass of the vimentin coil 1B in a solution was measured to equal 25 kDa, which corresponds to the dimeric structure [45]. The molecular masses of the vimentin rod, coil 1, and coil 2 fragments, previously determined by sedimentation equilibrium assay, correspond predominantly to the dimeric state of these coiled-coil regions [33]. However, for the GFAP 1B domain, both dimeric and tetrameric forms were found in a solution, with a predominant tetramer formation [46]. The results obtained on the isolated chicken desmin rod domain also support the ability of IF domains to self-assemble into different forms depending on ionic strength and protein concentration. The chicken desmin rod domain was present in a solution as a mixture of dimers and tetramers. In plain Tris buffer (pH 8.0), it formed predominantly dimers, while at higher ionic strengths, tetramers were also identified [47]. Taking into account our results and data from the literature, we assume that using the concentrations of 1–2 mg/mL of the NFL fragments in CD and DSC experiments we studied the melting of NFL_1B_, NFL_1A+1B_, and NFL_2_ dimeric structures. It is important to mention that NFL_FL,_ NFL_1B+2_, and NFL_ROD_ in the same experimental conditions are tetramer. Therefore, we can conclude that the thermal transitions in the NFL_FL_ melting curve, which have correspond with the thermal transitions of the NFL fragments (Table 3), reflect the denaturation of coiled-coil structures.

Among all the NFL fragments analyzed in this study, only NFL_2_ displayed irreversible thermal denaturation (Figure 7), while other proteins containing coil 2, namely NFL_1B+2_ and NFL_ROD_, underwent fully reversible thermal unfolding (Figure 3A,B, Figure 4B, Figure 5B and Figure 6C). Importantly, the refolding of NFL_2_ from the solution containing chaotropic agents differs from that after thermal denaturation (Figure 7A). Previously, it was elucidated that the end part of vimentin coil 2, which was stabilized on its C-end, might demonstrate the irreversible unfolding, while the same region, which was stabilized on its N-end, refolded successfully [30]. By using DSC with two-step heating, we have shown that among the two thermal transitions (at 37.2 °C and 62.7 °C), the second one (at 62.7 °C) is responsible for the irreversible denaturation (Figure 7D and Figure S1). Summing up all these data, we suggest that the N-terminal part of NFL coil 2 is the unstable region, and for proper folding, it mostly requires coil 1B.

At present, there is no doubt that the structure of the IF rod domain is α-helical with several segmented domains that form coiled-coil structures, and several α-helical linker regions. The first linker L1 connects the coil 1A and coil 1B domains, and the second linker L12 connects coil 1B and coil 2. In early predictions, there also was another linker—L2, which was suggested to connect coil 2A and 2B segments. However, the latest evidence stated that vimentin coil 2 has a continuous α-helical structure without any linkers inside [40]. However, the coil 2 structure still has some parts that differ in their thermal stability [30]. In our study, we showed that NFL coil 2 thermal denaturation has at least two thermal transitions, which occur at 37.2 and 62.7 °C (Figure 7D). Variable stability of coil 2 was also observed for the vimentin molecule by the single-molecule force spectroscopy with optical tweezers. It was shown that the coil 2B C-terminal part is more stable than the N-terminal and central part of it [48].

These facts provide some evidence that the heterogeneous stability of the IF’s coil 2 structure might be important for coil 2’s role in the IF’s dimer and tetramer self-assembly. The other region in IF that has lower thermal stability is coil 1A [29]. It is important for the longitudinal polymerization of IF while paired with the coil 2 C-terminus, as well as for lateral interactions while it is paired with the N-terminal part of coil 2 [49].

Despite the present data showing that coil 2 does not have a linker sequence inside, it differs from a traditional coiled-coil structure based on the heptad repeats of amino-acid residues. The hendecad repeats (*abcdefghijk*)_n_ of amino-acid residues were found in the conservative region at the beginning of coil 2 (first 36 residues) [17,40,41]. Furthermore, coil 2 of all IF members has a so-called “stutter”, which is located at the position of 8.5 heptads downstream of the beginning of coil 2 and corresponds to a four-residue insertion [14,41,42].

In the structure of a full NFL molecule, the appearance of mutation causes changes in its thermal stability. The Glu90Lys substitution that correlates with Charcot–Mary–Tooth neuropathy leads to the disappearance of the low-temperature thermal transition (first calorimetric domain on the DSC profile). More precisely, the NFL Glu90Lys has only three calorimetric domains at 41.6, 52.2, and 62.6 °C, and lacks thermal transition at 33.2 °C [32]. Comparing the data obtained on NFL WT (wild type) and its Glu90Lys mutant, we might suggest that calorimetric domain 1 on the DSC profile of NFL WT (NFL_FL_ in Figure 4A) corresponds to coil 1A denaturation. 

Summing up all the data for NFL_FL_ and its fragments’ thermal stability (Figure 4, Figure 6 and Figure 7D), we conclude that calorimetric domain 1 of NFL_FL_ yields to coil 1A denaturation, domain 2 corresponds to coil 1B, while the calorimetric domain 4 originates from coil 2 denaturation. The calorimetric domain 3 (at 53.6 °C) of the full-length NFL tetramer did not correspond to any fragments used in this work. Since the full-length NFL differs from NFL_ROD_ by the presence of N- and C-terminal parts (Figure 1), one can propose that this domain reflects the thermal unfolding of these parts in the NFL molecule. It might indicate that this thermal transition appears due to another molecular interaction that occurs in a full tetramer.

## 5. Conclusions

In the present work, we provided a detailed study of the thermal stability of neurofilament light protein by using a set of recombinant fragments that correspond to NFL coiled-coil domains. The thermal stability of coiled-coil domains of the IFs molecule plays an important role in their self-association. From our data, we can conclude that thermal transitions of isolated coiled-coil domains correlate well with thermal transitions found in the full-length NFL molecule. In particular, we identified the denaturation profiles of NFL coil 1B, coil 1A, and coil 2. Importantly, we demonstrated that NFL coil 2 has two thermal transitions. Moreover, we found that among all NFL domains, only coil 2 undergoes irreversible heat-induced denaturation as an isolated fragment. Meanwhile, being in a fusion with coil 1B or as a part of the full rod domain, the denaturation of coil 2 is fully reversible. We conclude that the N-terminal part of NFL coil 2 is the unstable region that requires coil 1B for proper folding.

## Figures and Tables

**Table 1:** Truncated NFL proteins studied in the current work.

Short Name of the Fragment	Name of the Structural Domain	Borders of the Truncated Fragment
NFL_1B_	Coil 1B	Ser133—Ile243
NFL_2_	Coil 2	Val249—Glu396
NFL_1B+2_	Coils 1B and 2	Ser133—Glu396
NFL_1A+1B_	Coils 1A and 1B	Ser80—Ile243
NFL_ROD_	Coils 1A, 1B, and 2	Ser80—Glu396

**Table 2:** Sequence of primers used to obtain truncated NFL proteins.

Primer	Sequence
FW80	5′-ATATATATCATATGCATCACCATCACCATCACCTGGAAGTGCTGTTTCAGGGCCCGAGCAACGACCTCAAGTCCATCCG-3′
FW133	5′-ATATATATCATATGCATCACCATCACCATCACCTGGAAGTGCTGTTTCAGGGCCCGTCCCGCTTCCGGGCGCT-3′
FW249	5′-ATATATATCATATGCATCACCATCACCATCACCTGGAAGTGCTGTTTCAGGGCCCGGTGACCAAGCCCGACCTTTCC-3′
REV243	5′-ATATATATGAATTCTTAGATCTGCGCGTACTGGATCTGCG-3′
REV396	5′-ATATATATGAATTCTTACTCCCCCTCCAGCAGTTTACGG-3′

**Table 3:** Calorimetric parameters * obtained by DSC for the individual thermal transitions (calorimetric domains) of the NFL fragments.

Sample	Number of Domain	T_m_^#^ (°C)	ΔH_cal_^§^ (kJ mol^−1^)	ΔH_cal_ (% of Total ΔH_cal_)	Total ΔH_cal_ (kJ mol^−1^)
NFL_1B_	Domain 1	39.4	165	100	165
NFL_1A+1B_	Domain 1	41.9	80	100	80
NFL_1B+2_	Domain 1 Domain 2 Domain 3	37.8 41.5 59.6	30 195 190	7 47 46	415
NFL_ROD_	Domain 1 Domain 2 Domain 3	33.7 41.8 61.1	35 205 155	9 52 39	395
NFL_FL_	Domain 1 Domain 2 Domain 3 Domain 4	33.2 41.7 53.6 63.2	15 245 215 115	3 42 36 19	590
NFL_2_	Domain 1 Domain 2	37.2 62.7	ND	ND	160

* Parameters were obtained from the data presented in Figure 4, Figure 6 and Figure 7. ^#^ Error of the given temperature values of the calorimetric domains (T_m_) did not exceed ±0.2 °C. ^§^ Relative error of the given values of the calorimetric enthalpy (ΔH_cal_) did not exceed 10%.

## Data Availability

The original data of the study are presented in the article and Supplementary Materials; further inquiries can be directed to the corresponding author.

## Supplementary Materials

The following supporting information can be downloaded at: https://www.mdpi.com/article/10.3390/biom14010085/s1, Figure S1: Temperature dependence of heat balance (in µW) of NFL_2_ protein obtained by DSC. Table S1: The liquid flow program used for the AF4 platform.

## References

[1] Kornreich M., Avinery R., Malka-Gibor E., Laser-Azogui A., Beck R. (2015). Order and disorder in intermediate filament proteins. FEBS Lett..

[2] Eldirany S.A., Lomakin I.B., Ho M., Bunick C.G. (2020). Recent insight into intermediate filament structure. Curr. Opin. Cell Biol..

[3] Etienne-Manneville S. (2018). Cytoplasmic Intermediate Filaments in Cell Biology. Annu. Rev. Cell Dev. Biol..

[4] Vermeire P.-J., Stalmans G., Lilina A.V., Fiala J., Novak P., Herrmann H., Strelkov S.V. (2021). Molecular interactions driving intermediate filament assembly. Cells.

[5] Burkhard P., Stetefeld J., Strelkov S.V. (2001). Coiled coils: A highly versatile protein folding motif. Trends Cell Biol..

[6] Mücke N., Wedig T., Bürer A., Marekov L.N., Steinert P.M., Langowski J., Aebi U., Herrmann H. (2004). Molecular and biophysical characterization of assembly-starter units of human vimentin. J. Mol. Biol..

[7] Wickert U., Mücke N., Wedig T., Müller S.A., Aebi U., Herrmann H. (2005). Characterization of the in vitro co-assembly process of the intermediate filament proteins vimentin and desmin: Mixed polymers at all stages of assembly. Eur. J. Cell Biol..

[8] Soellner P., Quinlan R.A., Franke W.W. (1985). Identification of a distinct soluble subunit of an intermediate filament protein: Tetrameric vimentin from living cells. Proc. Natl. Acad. Sci. USA.

[9] Eldirany S., Ho M., Hinbest A.J., Lomakin I.B., Bunick C.G. (2019). Human keratin 1/10-1B tetramer structures reveal a knob-pocket mechanism in intermediate filament assembly. EMBO J..

[10] Herrmann H., Haner M., Brettel M., Ku N.O., Aebi U. (1999). Characterization of distinct early assembly units of different intermediate filament proteins. J. Mol. Biol..

[11] Nakamura Y.Y., Takeda M., Angelides K.J., Tanaka T., Tada K., Nishimura T. (1990). Effect of phosphorylation on 68 KDa neurofilament subunit protein assembly by the cyclic AMP dependent protein kinase in vitro. Biochem. Biophys. Res. Commun..

[12] Yates D.M., Manser C., De Vos K.J., Shaw C.E., McLoughlin D.M., Miller C.C. (2009). Neurofilament subunit (NFL) head domain phosphorylation regulates axonal transport of neurofilaments. Eur. J. Cell. Biol..

[13] Hisanaga S., Gonda Y., Inagaki M., Ikai A., Hirokawa N. (1990). Effects of phosphorylation of the neurofilament L protein on filamentous structures. Cell Regul..

[14] Ahn J., Jeong S., Kang S.-M., Jo I., Park B.-J., Ha N.-C. (2021). Separation of coiled-coil structures in lamin A/C is required for the elongation of the filament. Cells.

[15] Turgay Y., Eibauer M., Goldman A.E., Shimi T., Khayat M., Ben-Harush K., Dubrovsky-Gaupp A., Sapra K.T., Goldman R.D., Medalia O. (2017). The molecular architecture of lamins in somatic cells. Nat. Cell Biol..

[16] Lee C.-H., Kim M.-S., Chung B.M., Leahy D.J., Coulombe P.A. (2012). Structural basis for heteromeric assembly and perinuclear organization of keratin filaments. Nat. Struct. Mol. Biol..

[17] Nicolet S., Herrmann H., Aebi U., Strelkov S.V. (2010). Atomic structure of vimentin coil 2. J. Struct. Biol..

[18] Chernyatina A.A., Nicolet S., Aebi U., Herrmann H., Strelkov S.V. (2012). Atomic structure of the vimentin central α-helical domain and its implications for intermediate filament assembly. Proc. Natl. Acad. Sci. USA.

[19] Bollati M., Barbiroli A., Favalli V., Arbustini E., Charron P., Bolognesi M. (2012). Structures of the lamin A/C R335W and E347K mutants: Implications for dilated cardiolaminopathies. Biochem. Biophys. Res. Commun..

[20] Ruan J., Xu C., Bian C., Lam R., Wang J.P., Kania J., Min J.R., Zang J.Y. (2012). Crystal structures of the coil 2B fragment and the globular tail domain of human lamin B1. FEBS Lett.

[21] Leterrier C., Dubey P., Roy S. (2017). The nano-architecture of the axonal cytoskeleton. Nat. Rev. Neurosci..

[22] Laser-Azogui A., Kornreich M., Malka-Gibor E., Beck R. (2015). Neurofilament assembly and function during neuronal development. Curr. Opin. Cell Biol..

[23] Athlan E.S., Mushynski W.E. (1997). Heterodimeric associations between neuronal intermediate filament proteins. J. Biol. Chem..

[24] Garden M.J., Eagles P.A. (1986). Chemical cross-linking analyses of ox neurofilaments. Biochem. J..

[25] Stone E.J., Uchida A., Brown A. (2019). Charcot–Marie–Tooth disease type 2E/1F mutant neurofilament proteins assemble into neurofilaments. Cytoskeleton.

[26] Adebola A.A., Gastri T.D., He C.-Z., Salvatierra L.A., Zhao J., Brown K., Lin C.-S., Worman H.J., Liem R.K.H. (2014). Neurofilament light polypeptide gene N98S mutation in mice leads to neurofilament network abnormalities and a Charcot–Marie–Tooth type 2E phenotype. Hum. Mol. Genet..

[27] Perez-Olle R., Jones S.T., Liem R.K.H. (2004). Phenotypic analysis of neurofilament light gene mutations linked to Charcot–Marie–Tooth disease in cell culture models. Hum. Mol. Genet..

[28] Jordanova A., De Jonghe P., Boerkoel C.F., Takashima H., De Vriendt E., Ceuterick C., Martin J.-J., Butler I.J., Mancias P., Papasozomenos S.C. (2003). Mutations in the neurofilament light chain gene (NEFL) cause early onset severe Charcot–Marie–Tooth disease. Brain.

[29] Meier M., Padilla G.P., Herrmann H., Wedig T., Hergt M., Patel T.R., Stetefeld J., Aebi U., Burkhard P. (2009). Vimentin coil 1A—A molecular switch involved in the initiation of filament elongation. J. Mol. Biol..

[30] Lilina A.V., Leekens S., Hashim H.M., Vermeire P.-J., Harvey J.N., Strelkov S.V. (2022). Stability profile of vimentin rod domain. Protein Sci..

[31] Brennich M.E., Vainio U., Wedig T., Bauch S., Herrmann H., Köster S. (2019). Mutation-induced alterations of intra-filament subunit organization in vimentin filaments revealed by SAXS. Soft Matter.

[32] Nefedova V.V., Yampolskaya D.S., Kleymenov S.Y., Chebotareva N.A., Matyushenko A.M., Levitsky D.I. (2023). Effect of neurodegenerative mutations in the NEFL gene on thermal denaturation of the neurofilament light chain protein. Biochemistry.

[33] Strelkov S.V., Herrmann H., Geisler N., Lustig A., Ivaninskii S., Zimbelmann R., Burkhard P., Aebi U. (2001). Divide-and-conquer crystallographic approach towards an atomic structure of intermediate filaments. J. Mol. Biol..

[34] Guzenko D., Chernyatina A.A., Strelkov S.V. (2017). Crystallographic studies of intermediate filament proteins. Subcell. Biochem..

[35] Nefedova V.V., Sudnitsyna M.V., Gusev N.B. (2017). Interaction of small heat shock proteins with light component of neurofilaments (NFL). Cell Stress Chaperones.

[36] Miles A.J., Ramalli S.G., Wallace B.A. (2022). DichroWeb, a website for calculating protein secondary structure from circular dichroism spectroscopic data. Protein Sci..

[37] Freire E., Biltonen R.L. (1978). Statistical mechanical deconvolution of thermal transitions in macromolecules. I. Theory and application to homogeneous systems. Biopolymers.

[38] Safenkova I.V., Slutskaya E.S., Panferov V.G., Zherdev A.V., Dzantiev B.B. (2016). Complex analysis of concentrated antibody-gold nanoparticle conjugates’ mixtures using asymmetric flow field-flow fractionation. J. Chromatogr. A.

[39] Strelkov S.V., Herrmann H., Aebi U. (2003). Molecular architecture of intermediate filaments. Bioessays.

[40] Chernyatina A.A., Strelkov S.V. (2012). Stabilization of vimentin coil2 fragment via an engineered disulfide. J. Struct. Biol..

[41] Herrmann H., Aebi U. (2016). Intermediate Filaments: Structure and Assembly. Cold Spring Harb. Perspect. Biol..

[42] Herrmann H., Aebi U. (2004). Intermediate filaments: Molecular structure, assembly mechanism, and integration into functionally distinct intracellular Scaffolds. Annu. Rev. Biochem..

[43] Burstein E.A., Vedenkina N.S., Ivkova M.N. (1973). Fluorescence and the location of tryptophan residues in protein molecules. Photochem. Photobiol..

[44] Geisler N., Heimburg T., Schunemann J., Weber K. (1993). Peptides from the conserved ends of the rod domain of desmin disassemble intermediate filaments and reveal unexpected structural features: A circular dichroism, Fourier transform infrared, and electron microscopic study. J. Struct. Biol..

[45] Vermeire P.-J., Lilina A.V., Hashim H.M., Dlabolová L., Fiala J., Beelen S., Kukačka Z., Harvey J.N., Novák P., Strelkov S.V. (2023). Molecular structure of soluble vimentin tetramers. Sci. Rep..

[46] Kim B., Kim S., Jin M.S. (2018). Crystal structure of the human glial fibrillary acidic protein 1B domain. Biochem. Biophys. Res. Commun..

[47] Potschka M., Nave R., Weber K., Geisler N. (1990). The two coiled coils in the isolated rod domain of the intermediate filament protein desmin are staggered A hydrodynamic analysis of tetramers and dimers. Eur. J. Biochem..

[48] Ramm B., Stigler J., Hinczewski M., Thirumalai D., Herrmann H., Woehlke G., Rief M. (2014). Sequence-resolved free energy profiles of stress-bearing vimentin intermediate filaments. Proc. Natl. Acad. Sci. USA.

[49] Premchandar A., Mücke N., Poznański J., Wedig T., Kaus-Drobek M., Herrmann H., Dadlez M. (2016). Structural dynamics of the vimentin coiled-coil contact regions involved in filament assembly as revealed by hydrogen-deuterium exchange. J. Biol. Chem..

