# Supplementary material for: Neurofilament Light Protein Rod Domain Exhibits Structural Heterogeneity
Source: Biomolecules. 2024 Jan 9;14(1):85. doi: 10.3390/biom14010085 (PMC10813002; doi:10.3390/biom14010085)
Supplement: Supplementary file 1 [file biomolecules-14-00085-s001.zip › biomolecules-2731920-supplementary.pdf]

Supplementary file

Table S1. The liquid flow program used for AF4 platform.

Start Time (min)	End Time (min)	Duration (min)	Mode	Vx Start (mL/min)t	Vx End (mL/min)
0.00	2.00	2.00	Elution	5.00	5.00
2.00	3.00	1.00	Focus		
3.00	4.00	1.00	Focus + Injection		
4.00	5.00	1.00	Focus		
5.00	10.00	5.00	Elution	5.00	5.00
10.00	12.00	2.00	Elution	5.00	4.00
12.00	17.00	5.00	Elution	4.00	4.00
17.00	25.00	8.00	Elution	4.00	0.10
25.00	35.00	10.00	Elution	0.10	0.00
35.00	38.00	3.00	Elution	5.00	5.00

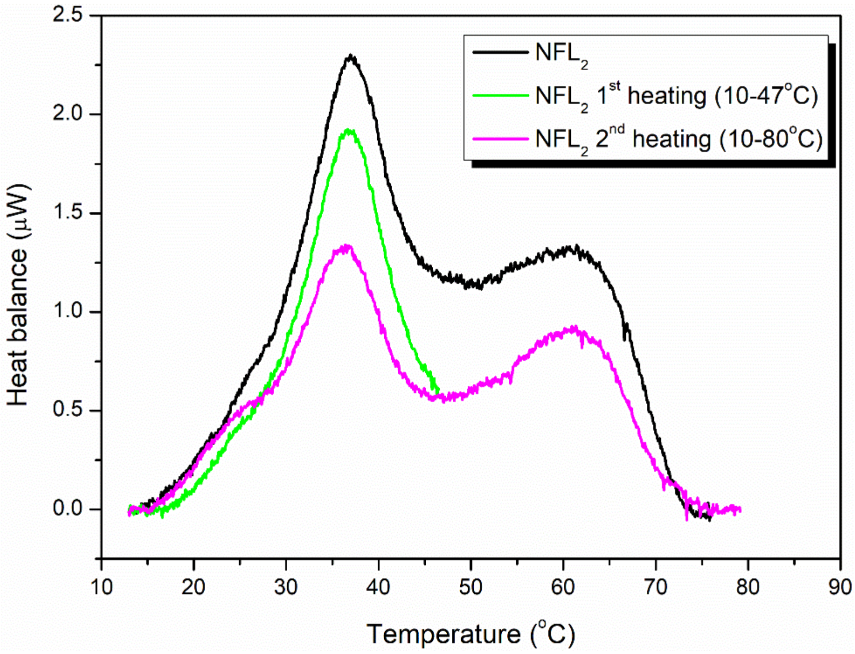

Figure S1. Temperature dependence of heat balance (in μW) of the NFL_2 protein obtained by DSC. Experimental DSC curves (without subtraction of instrumental and chemical baselines) are shown. The black line represents the denaturation of native NFL_2 protein heated from 10 to 85 °C. The green line represents the heating of NFL_2 protein from 10 to 47 °C («pre-heated»). This sample was cooled in the DSC cell and subjected to second heating (magenta line).
